# The use of HEPES-buffer in the production of gallium-68 radiopharmaceuticals – time to reconsider strict pharmacopoeial limits?

**DOI:** 10.1186/s41181-021-00129-w

**Published:** 2021-04-01

**Authors:** Jannie le Roux, Janke Kleynhans, Sietske Rubow

**Affiliations:** 1 NuMeRI Node for Infection Imaging, Central Analytical Facilities, Stellenbosch University, Stellenbosch, South Africa; 2 Nuclear Medicine Division, Stellenbosch University, Stellenbosch, South Africa

**Keywords:** HEPES, Gallium-68, Radiopharmaceuticals, Limits, Toxicity, Human use

## Abstract

HEPES (4-(2-hydroxyethyl) piperazine-1-ethanesulfonic acid) is a buffer that is used in the radiolabelling of gallium-68 compounds. The beneficial effects of HEPES on molar activity in bioconjugates have been well described. Current strict regulations on the HEPES content in radiopharmaceuticals limit its use when intended for parenteral administration.

This short communication summarizes data from the literature on the toxicity of HEPES in dogs after intravenous infusion and the subsequent use in humans. We also highlight the use of HEPES in an FDA labelled intravenous drug formulation. Regulatory institutions may consider this data to review current strict limits.

## Use of HEPES in pharmaceutical products

Radiosynthesis of gallium-68 compounds often requires a labelling mixture with a pH of 3.5–5.0 to ensure good complexation of gallium-68 with the precursor. Buffers such as 4-(2-hydroxyethyl) piperazine-1-ethanesulfonic acid (HEPES) and sodium acetate are extensively used in the labelling of radiopharmaceuticals with gallium-68. These buffers are reported to have low metallic complexation properties making them suitable to adjust pH during labelling and reduce formation of colloids (Bauwens et al. 2010).

HEPES, a zwitterionic buffer, is listed as a Good’s buffer with a pKa of 3.0 and 7.55 (Good et al. 1966). Velikyan et al. illustrated the beneficial effect of HEPES on specific activity in radiolabelling bioconjugates (Velikyan et al. 2004). A number of references indicate that adjusting the pH with HEPES during radiolabelling with gallium-68 provides optimum results in terms of molecular activity, reproducibility, reliability and versatility (Bauwens et al. 2010; Eppard et al. 2014; Pfaff et al. 2018; Velikyan et al. 2004). The European Pharmacopoeia prescribes a strict limit of 200 μg per dose for the HEPES content in radiopharmaceuticals intended for intravenous administration (European Pharmacopeia 2017). However, the method described in the European Pharmacopoeia to test for HEPES in radiopharmaceutical preparations has proven to be unreliable (Antunes et al. 2020; Pfaff et al. 2018; Sasson et al. 2010). The low limit has hampered the use of HEPES in gallium-68 radiopharmaceuticals, despite its superior buffering properties. A lack of toxicological data, especially after intravenous administration is generally cited as the main reason for these strict limits (Bauwens et al. 2010; European Pharmacopeia 2017; Sasson et al. 2010).

A chronic tolerance study of 4-(2-hydroxyethyl) piperazine-1-ethanesulfonic acid (product code TVZ-7), published in 1997 by Theodore et al.*,* provides important data on HEPES toxicity in beagle dogs after intravenous infusion (Theodore et al. 1997a). The animals were monitored for pharmacological or toxicological effects from increasing HEPES doses of 5–500 mg/kg, administered over a period of 148 days. Initially doses were administered daily via intravenous infusion but then changed to alternate days because of a subjective observation of stress at doses reaching 300, 350 and 400 mg/kg. Routine clinical pathology evaluations consisted of complete blood count and blood chemistry. Histopathology (bone marrow and liver biopsies) was done at the end of the highest dose segment when clinical effects were observed. Important results from the study included the following:

- Vomiting, a pharmacological effect of HEPES administration, occurred in the first two segments of the study but subsided when HEPES was administered before feeds.
- When I.V. doses approached 400 mg/kg (4000 mg for a dog with an average weight of 10 kg), significant changes in hematopoietic and reticuloendothelial system were observed in some dogs. These included hypercellular bone marrow and extramedullary hematopoiesis.

The study however concluded that no serious adverse effects are associated with chronic intravenous administration of HEPES in doses ranging from 5 to 500 mg/kg. In a patient with an average weight of 70 kg, this would amount to a maximum dose of 35,000 mg (35 g).

A more significant publication is the preliminary evaluation of a fixed dose of HEPES in humans by Theodore et al. The investigators report on the use of HEPES to evaluate its potential beneficial effects in clinical cancer (Theodore et al. 1997b). In this study, most subjects received an average fixed daily dose of 5000 mg, administered intravenously for 2 weeks. Thereafter, the same dose was administered three times per week for 2 more weeks. Patient response was evaluated at 4 weeks. A maintenance dose of 5000 mg, administered two to three times per week, was thereafter instituted. This study reported minimal side-effects and toxicity, similar to what was reported in the study with dogs.

The current limit for HEPES in radiopharmaceutical preparations specified by the European Pharmacopoeia. is 200 μg/V, where V represents the maximum injected dose in millilitre. This very strict limit translates to a maximum HEPES dose of 200 μg per dose. Labelling of specific radiopharmaceuticals may require different volumes and concentrations of HEPES as reported by Antunes et al. (Antunes et al. 2020).Using these examples, in a worst-case scenario where no HEPES is removed during the labelling process, a total single dose between 283 and 714 mg HEPES would be intravenously administered to a patient. This translates to a HEPES dose of 4.0–10.2 mg/kg for a 70 kg patient. The highest single dose is much lower than the maximum mg/kg dose used in the chronic toxicity study in dogs. This is approximately 14% of the average dose administered to humans in the preliminary fixed dose study by Theodore et al. It is known that a considerable amount of HEPES is removed during the purification process. The HEPES content is therefore significantly lower than the doses used in the Theodore toxicity study and still far below the chronic dose of 400 mg/kg where significant changes in the hematopoietic and reticuloendothelial systems were observed.

In 2015 the FDA approved ONIVYDE™ (irinotecan, Merrimack Pharmaceuticals), a topoisomerase 1 inhibitor which also contains HEPES as a buffering system. The pharmaceutical formulation contains 4.05 mg/ml HEPES in a 10 ml vial solution, to be diluted in 500 ml dextrose 5% for intravenous administration (Baker and Levien 2017; Merrimack 2015; Theodore et al. 1997a). The recommended dosage is 70 mg/m^2^ irinotecan (4.3 mg/ml) resulting in an average male (1.9 m^2^) receiving a total amount of 133 mg of the drug and 126 mg of HEPES. This amount of administered HEPES is much higher than the recommended 200 μg per dose for gallium-68 based radiopharmaceuticals. Limiting the HEPES content to 200 μg/V as currently advised by the European Pharmacopoeia may therefore be a far too strict limit.

The authors are aware that the studies reported are designed to evaluate the use of HEPES as an active pharmaceutical ingredient or as an adjuvant/excipient for therapeutic anticancer formulations. Further toxicological studies in animals or humans may be required to increase the European Pharmacopoeia limit for HEPES as an adjuvant for radiopharmaceutical use.

To conclude: The expected HEPES content in radiopharmaceutical preparations is significantly less than the doses used in the chronic toxicity study in dogs by Theodore et al. and in the other FDA approved pharmaceutical. The HEPES content is also considerably less that the dose used in humans to evaluate its effects in clinical cancer. It is further highly unlikely that patients scheduled for Nuclear Medicine investigations will receive a daily intravenous dose of 5000 mg HEPES dose as in the Theodore study. Taking these factors into account, regulatory institutions may consider reviewing the current strict limit for HEPES in radiopharmaceutical preparations.

## Data Availability

Not applicable.
